# Factors associated with gaps in naloxone knowledge: evidence from a 2022 great plains survey

**DOI:** 10.1186/s12954-024-00954-7

**Published:** 2024-02-10

**Authors:** Spencer Cooper-Ohm, Patrick Habecker, Ryan Humeniuk, Rick A. Bevins

**Affiliations:** 1 Department of Economics, University of Iowa, Iowa City, USA; 2 Rural Drug Addiction Research Center, University of Nebraska-Lincoln, Oldfather Hall – 4th Floor, 660 N 12th Street, Lincoln, NE 68588 USA; 3 Honors Tutorial College, Ohio University, Athens, USA; 4 Department of Psychology, University of Nebraska-Lincoln, Lincoln, USA

**Keywords:** Naloxone, Narcan®, Cascade of care, USA, Great plains, Harm reduction, Stigma

## Abstract

**Background:**

The rising prevalence of fast-acting opioids in the USA suggests the increased need for non-professional first responder administration of naloxone. Effective administration of naloxone during an overdose requires that bystanders are familiar with, have access to, and know how to use naloxone.

**Methods:**

Drawing on a statewide, address-based sample of Nebraskan adults, we used logistic regression to predict the likelihood of respondents’ familiarity with, access to, and competency to administer naloxone. Our independent variables included measures indicating proximity to drug use, perceived community stigma toward people who use drugs, and demographic data.

**Results:**

There were significant gaps in naloxone knowledge in Nebraska. Although 74.8% of respondents were familiar with naloxone, only 18.2% knew how to access it and 18.0% knew how to use it. Being close to an overdose experience, lifetime illicit opioid use, being close to a person who uses opioids, and having access to illicit opioids were not significantly associated with naloxone familiarity, access, or competency among respondents in Nebraska’s two largest cities, Omaha and Lincoln. Outside of these cities, being close to a past overdose experience and access to illicit opioids was associated with higher odds of naloxone access and competency, but lifetime opioid use and being close to a person who uses opioids were not. Finally, among those familiar with naloxone, a higher perception of community stigma toward people who use opioids generally was associated with lower odds of naloxone access and competency. Higher perception of community stigma toward people who use heroin, methamphetamines, and cocaine, however, was associated with higher odds of naloxone access.

**Conclusions:**

Our findings highlight the continued need for education on naloxone with a specific focus on access and competency to further reduce opioid-related overdose deaths. Specific focus should be placed on promoting naloxone knowledge among people with a higher likelihood of needing to administer naloxone to reduce otherwise avoidable deaths. Further work is needed to understand differences in the relationship between substance-specific perceived stigma and its association with naloxone access.

## Background

Rapid access to naloxone, an emergency medication that can reverse the effects of an ongoing opioid overdose, is a critical factor in preventing opioid-involved drug overdoses. Previously, naloxone distribution was primarily focused on professional first responders, medical professionals, and other professions that had high contact with overdose situations. As deaths associated with fast-acting synthetic opioids have increased since 2014, however, the distribution of naloxone to non-first responders to bypass emergency response delays has become more important. Naloxone possession is especially important among people who use drugs (PWUD) given their increased likelihood of witnessing an overdose: In 2014, over 80% of reported naloxone rescues were made by PWUD [26].

**Fig. 1:**
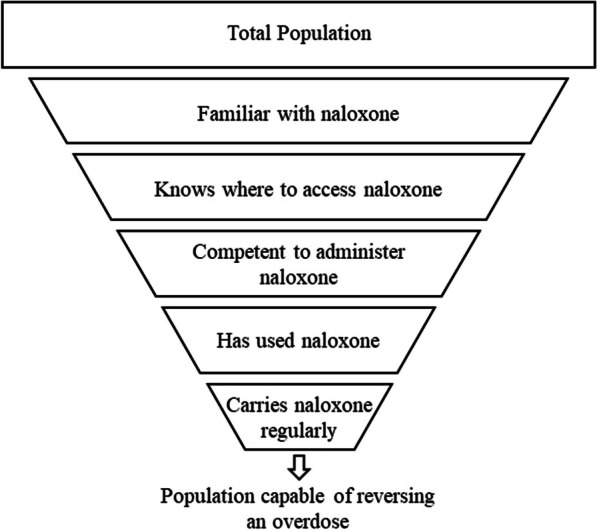
The naloxone treatment cascade

Attaining a high level of general population naloxone readiness requires a series of knowledge, access, and training goals. In short, people need to know naloxone exists, have access to naloxone, be trained to use naloxone, have used naloxone, and carry naloxone frequently (Fig. 1). These steps make up the “naloxone treatment cascade” [24]. Despite consistent annual nationwide increases in distribution of naloxone, there are currently significant gaps in the naloxone treatment cascade [7]. Studies find that although most surveyed adult Americans are familiar with naloxone, only a small proportion are aware that naloxone can be obtained in pharmacies [8, 21, 24]. Serious gaps in naloxone coverage also exist among PWUD: A 2021 meta-analysis of studies in areas across North America and Europe found that although 57% of PWUD owned naloxone, only 20–28% carried it on a regular basis [4].

Our study aims to expand on the literature surrounding naloxone awareness with a specific focus on the state of Nebraska’s unique place in the opioid epidemic by measuring its first three steps of the naloxone treatment cascade—naloxone familiarity, access, and competency to administer (referred to collectively as “naloxone knowledge”). Nebraska expanded public access to naloxone in May 2015 [15]. It currently has 112 pharmacies participating in the state’s free naloxone program and a free online service that ships Narcan to Nebraska addresses upon request [22]. Despite this progress, Nebraska’s network of naloxone distribution is still lacking in several respects. Nebraska is one of the few states that has outlawed syringe service programs (SSPs), which almost always facilitate opioid overdose education and naloxone distribution programs [11]. We are also unaware of any EMS naloxone leave behind programs or legislative efforts to facilitate naloxone distribution in criminal justice settings in the state. In 2019, Nebraska had the lowest per-capita pharmacy naloxone dispensation rate of all 50 states [7]. A prior study used a 2020 survey of Nebraskans to measure factors influencing naloxone access [20]. They estimated that 68.7% of respondents were familiar with naloxone and 15.0% knew where to access it, and using a multinomial logistic regression model, found that naloxone access was associated with having access to opioids and knowing someone who recently overdosed. We built on this research by creating new models to predict likelihood of naloxone familiarity and competency, using more recent data to observe patterns unfolding over time, and accounting for new variables in our model including the respondent’s perception of community stigma toward PWUD.

## Methods

Data for this project come from the Nebraska Annual Social Indicators Survey, an omnibus mail survey sent to an address-based sample of 8000 Nebraskan adults. In 2022, the sample frame was stratified evenly into the 6 behavioral health regions of the state, and a further 2 separately capture the two largest cities in the state, Omaha and Lincoln, which make up roughly 25% and 15% of the state’s population, respectively. Data were collected between July and November 2022. Full-sampling methodology and survey instruments are available through the University of Nebraska-Lincoln Bureau of Sociological Research [3].

A total of 1,455 completed or partially completed surveys were returned, for an AAPOR Response Rate 2 of 18.2% [1]. To account for the stratified sample design, data were weighed by stratum, within-household probability of selection, and non-response rate. Post-stratification weights were assigned based on region, age, and sex. 50.9% of survey responses had at least one missing value on measures in this paper, excluding forced skips. Missing values were estimated with 50 chained multiple imputations with the *mi* command suite in Stata 17 and a seed of 68,588.

Our primary dependent variables come from two questions on the survey. “*Do you know where to get Narcan (naloxone) if you needed it?”* (Yes/No/I don’t know what this is → Skip to next section) and *“Do you know how to use Narcan (naloxone)?”* (Yes/No). Respondents were categorized as having familiarity with naloxone if they answered “Yes” or “No” to the first question, and without familiarity if they answered “I don’t know what this is.” Naloxone access was determined by participants answering “Yes” to the first question. Finally, respondents were categorized as having naloxone competency if they responded “Yes” to the second question, and without competency if they answered “No” to the second question or responded “I don’t know what this is” to the first question and skipped the second question as instructed.

Perception of community stigma toward drug use was measured using an adaptation of the “awareness” portion of the brief opioid stigma scale [27]. Our scale used the average value of four questions on a 5-point Likert scale, with a value of 1 corresponding to “Strongly disagree” and 5 to “Strongly agree.” Respondents rated their agreement with the assertions that people in their community believe that a person who uses opioids “cannot be trusted,” is “dangerous,” “to blame for their own problems,” and “lazy.” The same questions were asked regarding a person who uses “cocaine, methamphetamines, and heroin.” While these categories overlap (heroin is an opioid), assessing community stigma in this fashion allowed respondents to differentiate between stigma toward opioids at large and stigma toward explicitly prohibited drugs. Other variables included yes/no answers to having used illicit opioids or heroin in their lifetime, being close with someone that currently used illicit opioids or heroin, knowing someone that experienced an overdose in the past year, having access to illicit opioids or heroin, and knowing what SSPs are.

The survey also collected information on age, sex, race, ethnicity, highest education obtained, household income, partner status, number of children present in the household, political orientation, religious affiliation, self-assessed rurality, and rurality based on Core-Based Statistical Areas. Due to sample size restrictions, we divide race and ethnicity into White non-Hispanic and non-White/Hispanic categories. Household income is divided from $0–$30,000, $30,001–$100,000, and $100,001+ . Respondents were asked how often they attended religious services with eight response options, ranging from “Several times a week” to “Never.” We reverse coded responses so 7 represents the highest attendance frequency and treated the resulting recode as a continuous variable.

We used logistic regression to predict likelihood of naloxone familiarity. Then, restricted to respondents with naloxone familiarity, we predicted respondents’ likelihood of naloxone access and naloxone competency. Then, we performed the same analysis for respondents located in Omaha and Lincoln and separately for respondents in all other regions. Analyses were conducted in Stata 17 with the *svy* command for sample design and weights, and *mi* commands for multiple imputation.

## Results

Table 1 shows descriptive statistics of our sample. The majority of respondents reported knowing what naloxone is (74.8%), but few knew where they could access naloxone (18.2%) or how to use it (18.0%). Among those with naloxone familiarity, 24.3% of respondents knew where to access naloxone and 24.01% knew how to use it. Few respondents knew someone who experienced an overdose in the past year (5.6%), were close to a person who uses illicit opioids or heroin (6.1%), or used illicit opioids or heroin in their lifetime (6.6%). 30.2% of respondents were familiar with SSPs. When we separated respondents in Omaha and Lincoln from the rest of the state, they were substantially more metropolitan (100% vs. 38.5%), more liberal (31.9% vs. 13.8%), and had less religious affiliation (26.3% had no affiliation vs. 18.6%).

**Table 1:** Descriptive statistics

Measure	Mean/Proportion	Standard error of estimate	95% Confidence interval of estimate		Mean/Proportion	Standard Error of Estimate	95% Confidence Interval of Estimate		Mean/Proportion	Standard Error of Estimate	95% Confidence Interval of Estimate	
(n = 1455, m = 50)												
	Total sample (n = 1455)				Omaha & Lincoln Only (n = 356)				Outside Omaha & Lincoln (n = 1099)			
Mean Age (years)	53.05	0.57	51.92	54.17	51.76	1.03	49.73	53.79	53.98	0.64	52.72	55.23
*Gender*												
Male	49.0%	1.9%	45.4%	52.7%	48.2%	3.5%	41.4%	55.0%	49.6%	2.0%	45.7%	53.5%
Female	51.0%	1.9%	47.4%	54.6%	51.8%	3.5%	45.0%	58.6%	50.4%	2.0%	46.5%	54.3%
*Race/Ethnicity*												
White non-Hispanic	90.5%	1.2%	88.1%	92.8%	12.5%	2.4%	7.8%	17.3%	7.4%	1.1%	5.2%	9.6%
Non-White/Hispanic	9.5%	1.2%	7.2%	11.9%	87.5%	2.4%	82.7%	92.2%	92.6%	1.1%	90.4%	94.8%
*Education*												
No College	17.7%	1.3%	15.1%	20.3%	11.9%	2.2%	7.5%	16.3%	21.9%	1.6%	18.8%	25.0%
Technical Degree/Some College	32.0%	1.7%	28.6%	35.3%	28.2%	3.2%	22.0%	34.4%	34.7%	1.9%	31.0%	38.4%
B.A./Terminal Degree	50.4%	1.8%	46.8%	53.9%	59.9%	3.4%	53.2%	66.6%	43.5%	2.0%	39.6%	47.3%
*Employment*												
Unemployed	29.9%	1.6%	26.8%	33.0%	29.5%	2.9%	23.7%	35.3%	30.2%	1.7%	26.9%	33.4%
Employed	70.1%	1.6%	67.0%	73.2%	70.5%	2.9%	64.7%	76.3%	69.8%	1.7%	66.6%	73.1%
*Annual Income*												
$0–$30,000	12.5%	1.1%	10.5%	14.6%	10.1%	1.9%	6.3%	13.9%	14.3%	1.2%	12.0%	16.5%
$30,001–$100,000	49.0%	1.9%	45.4%	52.7%	48.6%	3.5%	41.8%	55.4%	49.3%	2.0%	45.5%	53.2%
$100,001+	38.4%	1.9%	34.8%	42.1%	41.3%	3.4%	34.5%	48.0%	36.4%	2.0%	32.4%	40.3%
*Partner status*												
No partner	26.9%	1.6%	23.8%	29.9%	29.5%	3.0%	23.5%	35.4%	25.0%	1.6%	21.9%	28.1%
Married/With partner	73.1%	1.6%	70.1%	76.2%	70.5%	3.0%	64.6%	76.5%	75.0%	1.6%	71.9%	78.1%
*Political orientation*												
Liberal	21.4%	1.6%	18.3%	24.5%	31.9%	3.2%	25.6%	38.2%	13.8%	1.4%	11.0%	16.6%
Moderate	36.9%	1.9%	33.2%	40.6%	38.6%	3.5%	31.8%	45.4%	35.6%	2.0%	31.7%	39.6%
Conservative	41.8%	1.8%	38.2%	45.3%	29.5%	3.2%	23.2%	35.8%	50.6%	2.0%	46.7%	54.5%
*Religious denomination*												
Protestant	42.2%	1.8%	38.7%	45.7%	35.0%	3.2%	28.8%	41.2%	47.4%	2.0%	43.5%	51.4%
Catholic	24.2%	1.6%	21.1%	27.3%	26.8%	3.0%	20.8%	32.7%	22.3%	1.7%	19.1%	25.6%
No affiliation	21.8%	1.6%	18.6%	25.0%	26.3%	3.1%	20.1%	32.4%	18.6%	1.6%	15.4%	21.8%
Other	11.8%	1.3%	9.3%	14.3%	12.0%	2.4%	7.2%	16.7%	11.6%	1.4%	9.0%	14.3%
Mean religious attendance*	3.157	0.09	2.99	3.33	2.84	0.16	2.52	3.17	3.38	0.09	3.20	3.57
*Residence*												
Town or City	83.2%	1.1%	81.0%	85.4%	97.9%	0.9%	96.2%	99.7%	72.6%	1.7%	69.2%	75.9%
Open Country; not Farm	9.0%	0.9%	7.3%	10.7%	1.5%	0.7%	0.0%	2.9%	14.4%	1.4%	11.7%	17.2%
Farm	7.8%	0.8%	6.3%	9.3%	0.6%	0.6%	-0.5%	1.8%	13.0%	1.2%	10.6%	15.4%
*Core-Based Statistical Areas*												
Metropolitan	64.3%	1.1%	62.1%	66.5%	100.0%				38.5%	1.6%	35.4%	41.6%
Micropolitan	17.0%	1.0%	15.0%	19.0%	0.0%				29.2%	1.7%	25.9%	32.5%
Counties Outside	18.7%	1.0%	16.8%	20.6%	0.0%				32.3%	1.6%	29.1%	35.4%
*Close to past-year overdose?*												
No	94.4%	0.9%	92.6%	96.2%	94.4%	1.8%	90.9%	97.9%	94.4%	0.9%	92.7%	96.1%
Yes	5.6%	0.9%	3.8%	7.4%	5.6%	1.8%	2.1%	9.1%	5.6%	0.9%	3.9%	7.3%
*Lifetime opioid or heroin use?*												
No	93.4%	1.1%	91.4%	95.5%	93.5%	2.0%	89.5%	97.5%	93.3%	1.1%	91.3%	95.4%
Yes	6.6%	1.1%	4.5%	8.6%	6.5%	2.0%	2.5%	10.5%	6.7%	1.1%	4.6%	8.7%
*Close to opioid or heroin use?*												
No	93.9%	1.0%	91.9%	95.9%	91.6%	2.2%	87.4%	95.8%	95.6%	0.8%	94.0%	97.2%
Yes	6.1%	1.0%	4.1%	8.1%	8.4%	2.2%	4.2%	12.6%	4.4%	0.8%	2.8%	6.0%
*Access to opioids or heroin?*												
No	84.2%	1.4%	81.4%	87.0%	83.9%	2.7%	78.7%	89.2%	84.4%	1.5%	81.5%	87.4%
Yes	15.8%	1.4%	13.0%	18.6%	16.1%	2.7%	10.8%	21.3%	15.6%	1.5%	12.6%	18.5%
Mean community opioid stigma (5-point Likert Scale)	3.45	0.03	3.40	3.51	3.40	0.05	3.30	3.50	3.49	0.03	3.44	3.55
Mean community heroin, methamphetamine, and cocaine stigma (5-point Likert Scale)	3.67	0.02	3.62	3.71	3.59	0.04	3.51	3.68	3.72	0.03	3.67	3.77
*Familiar with SSPs?*												
No	69.8%	1.8%	66.2%	73.3%	61.5%	3.5%	54.7%	68.4%	75.7%	1.7%	72.3%	79.1%
Yes	30.2%	1.8%	26.7%	33.8%	38.5%	3.5%	31.6%	45.3%	24.3%	1.7%	20.9%	27.7%
*Familiar with naloxone?*												
No	25.2%	1.5%	22.3%	28.0%	18.1%	2.4%	13.5%	22.8%	30.2%	1.8%	26.7%	33.8%
Yes	74.8%	1.5%	72.0%	77.7%	81.9%	2.4%	77.2%	86.5%	69.8%	1.8%	66.2%	73.3%
*Access to naloxone?*												
No	81.8%	1.4%	79.0%	84.6%	81.0%	2.7%	75.7%	86.3%	82.3%	1.5%	79.3%	85.3%
Yes	18.2%	1.4%	15.4%	21.0%	19.0%	2.7%	13.7%	24.3%	17.7%	1.5%	14.7%	20.7%
*Competent to administer naloxone?*												
No	82.0%	1.5%	79.0%	85.0%	77.5%	3.0%	71.6%	83.5%	85.1%	1.4%	82.3%	87.9%
Yes	18.0%	1.5%	15.0%	21.0%	22.5%	3.0%	16.5%	28.4%	14.9%	1.4%	12.1%	17.7%
*Access to Naloxone among those Familiar (n = 1028)*												
No	75.7%	1.9%	72.0%	79.3%	76.9%	3.2%	70.5%	83.2%	74.7%	2.1%	70.6%	78.7%
Yes	24.3%	1.9%	20.7%	28.0%	23.1%	3.2%	16.8%	29.5%	25.3%	2.1%	21.3%	29.4%
*Competent to Administer Naloxone among those Familiar (n = 1028)*												
No	75.9%	2.0%	72.1%	79.8%	72.6%	3.6%	65.5%	79.7%	78.8%	2.0%	74.9%	82.6%
Yes	24.1%	2.0%	20.2%	27.9%	27.4%	3.6%	20.3%	34.5%	21.2%	2.0%	17.4%	25.1%

After survey weights, our sample had an above average proportion of White and educated respondents. Non-Hispanic White respondents made up 90.5% of our sample compared to 76.9% of the Nebraska population at large, and survey respondents with a bachelor’s degree or higher made up 50.4% of our sample compared to 39.2% in Nebraska [25].

Table 2 shows that familiarity with SSPs was associated with higher odds of familiarity with naloxone (OR = 2.292, *p* < 0.001). Having a technical degree or some college compared to no college education (OR = 1.881, *p* = 0.005), and an annual income above $100,001 compared to an income below $30,001 (OR = 1.939, *p* = 0.036) was associated with higher odds of naloxone familiarity. More frequent religious attendance was associated with lower odds of naloxone familiarity (OR = 0.894, *p* = 0.006).

**Table 2:** Logistic regression models predicting knowledge of naloxone (m = 50)

Measure	Naloxone Familiarity				Naloxone Access Among those with Familiarity				Naloxone Competency Among those with Familiarity			
	(n = 1455)				(n = 1028)				(n = 1028)			
	OR	*p*	95% Conf. interval		OR	*p*	95% conf. interval		OR	*p*	95% Conf. interval	
		Value				Value				Value		
Age (years)	1.000	0.951	0.988	1.013	*1.023*	*0.011*	*1.005*	*1.040*	1.001	0.941	0.981	1.021
*Gender*												
Male	ref				ref				ref			
Female	1.101	0.569	0.790	1.536	1.230	0.368	0.784	1.928	1.449	0.132	0.894	2.350
*Race/Ethnicity*												
White non-Hispanic	Ref				Ref				Ref			
Non-White/Hispanic	1.294	0.401	0.708	2.365	1.141	0.752	0.502	2.591	0.976	0.957	0.407	2.341
*Education*												
No College	Ref				Ref				Ref			
Technical Degree/Some College	*1.881*	*0.005*	*1.211*	*2.922*	1.093	0.809	0.531	2.247	1.270	0.532	0.600	2.691
B.A./Terminal Degree	1.509	0.086	0.943	2.414	1.358	0.394	0.672	2.748	1.184	0.645	0.576	2.435
*Employment*												
Unemployed	Ref				Ref				Ref			
Employed	0.894	0.575	0.603	1.325	*2.227*	*0.003*	*1.311*	*3.782*	1.655	0.160	0.819	3.344
*Annual income*												
$0–$30,000	Ref				Ref				Ref			
$30,001–$100,000	1.347	0.236	0.822	2.208	1.142	0.729	0.538	2.424	0.976	0.949	0.459	2.074
$100,001+	*1.939*	*0.036*	*1.045*	*3.595*	1.085	0.847	0.473	2.490	0.839	0.692	0.351	2.002
*Partner status*												
No partner	Ref				Ref				Ref			
Married/With Partner	1.266	0.217	0.870	1.840	1.292	0.327	0.774	2.156	1.328	0.295	0.781	2.259
*Political Orientation*												
Liberal	Ref				Ref				Ref			
Moderate	0.875	0.629	0.509	1.505	0.785	0.433	0.428	1.440	1.800	0.064	0.966	3.353
Conservative	0.620	0.083	0.362	1.065	0.730	0.326	0.389	1.368	1.357	0.388	0.678	2.715
*Religious denomination*												
Protestant	Ref				Ref				Ref			
Catholic	0.982	0.930	0.652	1.478	1.252	0.431	0.716	2.190	*1.905*	*0.033*	1.052	3.449
No affiliation	0.922	0.772	0.534	1.594	0.931	0.838	0.468	1.853	1.223	0.616	0.557	2.685
Other	0.893	0.690	0.513	1.555	1.356	0.407	0.660	2.784	1.091	0.839	0.470	2.530
Religious attendance*	*0.894*	*0.006*	*0.825*	*0.968*	0.975	0.645	0.877	1.084	0.960	0.507	0.850	1.084
*Residence*												
City or Town	Ref				Ref				Ref			
Open Country; not a Farm	1.016	0.953	0.605	1.705	1.180	0.634	0.597	2.333	0.943	0.871	0.463	1.919
Farm	0.744	0.280	0.435	1.273	1.141	0.730	0.540	2.410	0.729	0.471	0.308	1.725
*Core-based statistical areas*												
Metropolitan	Ref				Ref				Ref			
Micropolitan	1.001	0.997	0.662	1.514	1.247	0.422	0.727	2.140	0.849	0.559	0.490	1.471
Counties Outside	0.795	0.252	0.536	1.178	1.220	0.500	0.685	2.173	0.881	0.685	0.477	1.626
*Close to past-year overdose?*												
No	Ref				Ref				Ref			
Yes	0.595	0.181	0.278	1.274	*3.243*	*0.007*	*1.382*	*7.609*	*2.876*	*0.012*	*1.257*	*6.579*
*Lifetime Opioid or Heroin Use?*												
No	Ref				Ref				Ref			
Yes	1.049	0.920	0.412	2.673	0.726	0.476	0.301	1.750	1.107	0.825	0.448	2.737
*Close to Opioid or Heroin Use?*												
No	Ref				Ref				Ref			
Yes	1.546	0.403	0.557	4.291	0.543	0.238	0.196	1.499	0.415	0.115	0.139	1.238
*Access to Opioids or Heroin?*												
No	Ref				Ref				Ref			
Yes	1.021	0.937	0.605	1.726	*2.551*	*0.001*	*1.450*	*4.487*	1.754	0.069	0.957	3.213
Community Opioid Stigma (5-point Likert Scale)	0.874	0.402	0.637	1.198	*0.515*	*0.002*	*0.336*	*0.788*	0.671	0.092	0.421	1.067
Community Heroin, Methamphetamine, and Cocaine Stigma (5-point Likert Scale)	1.115	0.520	0.800	1.553	*1.884*	*0.009*	*1.172*	*3.028*	1.173	0.505	0.734	1.873
*Familiar with SSPs?*												
No	Ref				Ref				Ref			
Yes	*2.292*	* <0.001*	*1.487*	*3.533*	*2.530*	* < 0.001*	*1.608*	*3.979*	*4.325*	* < 0.001*	*2.640*	*7.087*
Constant	1.715	0.489	0.372	7.897	*0.016*	* < 0.001*	*0.002*	*0.154*	*0.091*	*0.053*	*0.008*	*1.032*

*Continuous recode of eight possible responses, ranging from 0 (never) to 7 (several times a week)

Restricting our analysis to respondents familiar with naloxone, analysis shows that familiarity with SSPs (OR = 2.53, *p* < 0.001), being employed compared to unemployed (OR = 2.227, *p* = 0.003), being close to a past-year overdose (OR = 3.243, *p* = 0.007), and having access to illicit opioids or heroin compared to not having access (OR = 2.551, *p* = 0.001) was associated with a higher likelihood of naloxone access. Higher perceived community stigma toward people who use opioids was associated with lower odds of naloxone access (OR = 0.515, *p* = 0.002), while higher perceived community stigma toward people who use heroin, methamphetamines, and cocaine was associated with higher odds of naloxone access (OR = 1.884, *p* = 0.009).

Among those familiar with naloxone, familiarity with SSPs (OR = 4.325, *p* < 0.001), being Catholic compared to Protestant (OR = 1.905, *p* = 0.033), being close to a past-year overdose (OR 2.876, *p* = 0.012) was associated with higher odds of naloxone competency.

Tables 3 and 4 replicate the analysis from Table 2 with two subpopulations: Omaha and Lincoln, the largest urban areas of the state (Table 3); and outside the these areas (Table 4). Urban and rural areas have notable differences in perceptions of stigma toward people who use drugs [2, 23], access to public services (e.g., rural areas may have longer EMS response times and fewer harm reduction organizations), and political views. Variables measuring respondents’ Core-Based Statistical Area and residence were dropped due to collinearity in these models.

**Table 3:** Logistic regression subgroup models predicting knowledge of naloxone in Omaha and Lincoln (m = 50)

Measure	Naloxone Familiarity				Naloxone access among those with familiarity				Naloxone competency among those with familiarity			
	(n = 356)				(n = 274)				(n = 274)			
	OR	*p*	95% Conf. interval		OR	*p*	95% Conf. Interval		OR	*p*	95% Conf. interval	
		Value				Value				Value		
Age (years)	0.976	0.052	0.953	1.000	1.031	0.088	0.995	1.067	0.985	0.402	0.951	1.020
*Gender*												
Male	Ref				Ref				Ref			
Female	1.115	0.761	0.552	2.251	1.094	0.829	0.484	2.471	1.726	0.189	0.763	3.903
*Race/Ethnicity*												
White non-Hispanic	Ref				Ref				Ref			
Non-White/Hispanic	0.670	0.449	0.237	1.892	0.880	0.843	0.248	3.119	0.994	0.993	0.270	3.666
*Education*												
No College	Ref				Ref				Ref			
Technical Degree/Some College	1.822	0.250	0.655	5.070	2.875	0.254	0.466	17.734	4.766	0.157	0.547	41.488
B.A./Terminal Degree	0.929	0.887	0.335	2.578	2.750	0.269	0.455	16.627	4.814	0.152	0.559	41.445
*Employment*												
Unemployed	Ref				Ref				Ref			
Employed	*0.398*	*0.027*	*0.176*	*0.902*	*4.980*	*0.008*	*1.519*	*16.329*	1.842	0.319	0.553	6.138
*Annual Income*												
$0–$30,000	Ref				Ref				Ref			
$30,001–$100,000	2.416	0.113	0.810	7.212	0.302	0.110	0.069	1.314	0.489	0.316	0.120	1.984
$100,001+	*5.264*	*0.017*	*1.350*	*20.530*	*0.146*	*0.022*	*0.028*	*0.759*	0.375	0.222	0.077	1.819
*Partner status*												
No Partner	Ref				Ref				Ref			
Married/with partner	1.200	0.639	0.559	2.576	1.512	0.377	0.603	3.790	1.117	0.813	0.445	2.802
*Political orientation*												
Liberal	Ref				Ref				Ref			
Moderate	0.443	0.115	0.160	1.223	0.575	0.274	0.213	1.553	*2.671*	*0.048*	*1.008*	*7.081*
Conservative	*0.336*	*0.034*	*0.123*	*0.918*	0.555	0.363	0.155	1.983	2.019	0.291	0.547	7.454
*Religious Denomination*												
Protestant	Ref				Ref				Ref			
Catholic	0.793	0.574	0.353	1.784	1.456	0.488	0.502	4.218	*3.426*	*0.019*	1.223	9.598
No Affiliation	0.489	0.220	0.156	1.536	1.546	0.502	0.431	5.542	1.472	0.585	0.366	5.918
Other	0.862	0.789	0.289	2.569	1.472	0.595	0.352	6.150	1.086	0.915	0.238	4.949
Religious Attendance*	*0.819*	*0.023*	*0.690*	*0.972*	1.023	0.827	0.835	1.253	0.897	0.344	0.716	1.124
*Close to Past-year Overdose?*												
No	Ref				Ref				Ref			
Yes	0.918	0.938	0.107	7.866	1.009	0.992	0.176	5.787	1.773	0.460	0.386	8.139
*Lifetime Opioid or Heroin Use?*												
No	Ref				Ref				Ref			
Yes	0.706	0.745	0.086	5.785	1.221	0.818	0.222	6.720	2.223	0.365	0.393	12.587
*Close to Opioid or Heroin Use?*												
No	Ref				Ref				Ref			
Yes	0.501	0.419	0.093	2.690	0.483	0.466	0.068	3.435	0.430	0.387	0.063	2.922
*Access to Opioids or Heroin?*												
No	Ref				Ref				Ref			
Yes	2.068	0.210	0.663	6.452	1.713	0.306	0.610	4.809	1.319	0.620	0.440	3.950
Community Opioid Stigma (5-point Likert Scale)	0.951	0.877	0.502	1.801	*0.435*	*0.039*	*0.197*	*0.959*	0.824	0.650	0.356	1.905
Community Heroin, Methamphetamine, and Cocaine Stigma (5-point Likert Scale)	1.169	0.633	0.615	2.223	*2.491*	*0.056*	*0.977*	*6.355*	0.812	0.616	0.359	1.838
*Familiar with SSPs?*												
No	Ref				Ref				Ref			
Yes	*3.771*	*0.003*	*1.559*	*9.119*	1.464	0.359	0.647	3.309	*3.600*	*0.003*	*1.543*	*8.399*
Constant	*26.238*	*0.037*	*1.217*	*565.893*	*0.011*	*0.021*	* < 0.001*	*0.500*	0.165	0.325	0.005	6.028

*Continuous recode of eight possible responses, ranging from 0 (never) to 7 (several times a week)

**Table 4:** Logistic regression subgroup models predicting knowledge of naloxone in regions outside Omaha and Lincoln (m = 50)

Measure	Naloxone Familiarity				Naloxone Access Among those with Familiarity				Naloxone Competency Among those with Familiarity			
	(n = 1,099)				(n = 754)				(n = 754)			
	OR	*p*	95% Conf. Interval		OR	*p*	95% Conf. Interval		OR	*p*	95% Conf. Interval	
		Value				Value				Value		
Age (years)	1.011	0.124	0.997	1.025	*1.021*	*0.037*	*1.001*	*1.042*	1.013	0.249	0.991	1.036
*Gender*												
Male	Ref				Ref				Ref			
Female	1.138	0.493	0.786	1.647	1.293	0.316	0.783	2.135	1.273	0.400	0.726	2.232
*Race/Ethnicity*												
White non-Hispanic	Ref				Ref				Ref			
Non-White/Hispanic	1.884	0.072	0.944	3.761	1.725	0.376	0.515	5.780	1.290	0.716	0.327	5.084
*Education*												
No College	Ref				Ref				Ref			
Technical Degree/Some College	*1.894*	*0.010*	*1.166*	*3.077*	0.638	0.230	0.306	1.330	0.699	0.368	0.320	1.526
B.A./Terminal Degree	*1.685*	*0.048*	*1.005*	*2.826*	0.852	0.667	0.411	1.767	0.534	0.119	0.243	1.175
*Employment*												
Unemployed	Ref				Ref				Ref			
Employed	1.251	0.321	0.803	1.947	1.602	0.119	0.886	2.900	1.744	0.159	0.804	3.782
*Annual income*												
$0–$30,000	Ref				Ref				Ref			
$30,001–$100,000	1.170	0.544	0.704	1.945	*2.242*	*0.042*	*1.032*	*4.871*	1.303	0.565	0.528	3.221
$100,001+	1.509	0.220	0.782	2.910	*3.390*	*0.005*	*1.444*	*7.959*	1.400	0.510	0.514	3.811
*Partner Status*												
No Partner	Ref				Ref				Ref			
Married/With Partner	1.304	0.203	0.867	1.961	1.359	0.294	0.765	2.413	1.687	0.111	0.887	3.210
*Political Orientation*												
Liberal	Ref				Ref				Ref			
Moderate	1.414	0.264	0.769	2.601	0.915	0.811	0.443	1.893	1.287	0.505	0.613	2.703
Conservative	0.922	0.790	0.505	1.683	0.825	0.584	0.413	1.645	0.899	0.792	0.408	1.984
*Religious Denomination*												
Protestant	Ref				Ref				Ref			
Catholic	1.029	0.903	0.652	1.622	1.190	0.585	0.638	2.221	1.237	0.551	0.614	2.488
No Affiliation	1.089	0.789	0.583	2.033	0.682	0.379	0.291	1.600	0.964	0.938	0.386	2.407
Other	0.913	0.775	0.488	1.707	1.353	0.481	0.583	3.138	1.131	0.817	0.398	3.218
Religious Attendance*	*0.911*	*0.041*	*0.833*	*0.996*	0.974	0.672	0.862	1.100	0.997	0.968	0.859	1.158
*Close to Past-year Overdose?*												
No	Ref				Ref				Ref			
Yes	*0.502*	*0.041*	*0.259*	*0.971*	*6.060*	* < 0.001*	*2.212*	*16.605*	*3.568*	*0.014*	*1.300*	*9.791*
*Lifetime Opioid or Heroin Use?*												
No	Ref				Ref				Ref			
Yes	1.348	0.505	0.560	3.246	0.470	0.145	0.170	1.299	0.765	0.604	0.278	2.105
*Close to Opioid or Heroin Use?*												
No	Ref				Ref				Ref			
Yes	2.464	0.070	0.928	6.541	0.797	0.653	0.295	2.152	0.524	0.222	0.186	1.479
*Access to Opioids or Heroin?*												
No	Ref				Ref				Ref			
Yes	0.983	0.953	0.552	1.750	*3.362*	*0.001*	*1.667*	*6.780*	*2.230*	*0.033*	*1.065*	*4.669*
Community Opioid Stigma (5-point Likert Scale)	0.843	0.367	0.581	1.223	*0.576*	*0.018*	*0.365*	*0.910*	*0.605*	*0.040*	*0.374*	*0.978*
Community Heroin, Methamphetamine, and Cocaine Stigma (5-point Likert Scale)	1.109	0.602	0.751	1.636	1.525	0.089	0.937	2.480	1.448	0.168	0.856	2.450
*Familiar with SSPs?*												
No	ref				ref				ref			
Yes	*1.813*	*0.017*	*1.114*	*2.949*	*4.574*	*< 0.001*	*2.749*	*7.610*	*6.649*	* < 0.001*	*3.826*	*11.557*
Constant	*0.340*	*0.237*	*0.057*	*2.036*	*0.012*	*0.003*	*0.001*	*0.212*	*0.024*	*0.024*	*0.001*	*0.616*

*Continuous recode of eight possible responses, ranging from 0 (never) to 7 (several times a week)

In Omaha and Lincoln (Table 3), being employed compared to unemployed was associated with lower odds of naloxone familiarity (OR = 0.398, *p* = 0.027), but among those with naloxone familiarity, being employed compared to unemployed was still associated with higher odds of naloxone access (OR = 4.980, *p* = 0.008) as it was in our statewide model. Among those familiar with naloxone in Omaha and Lincoln, higher perceived community stigma toward people who use opioids was associated with lower odds of naloxone access (OR = 0.435, *p* = 0.039). Identifying as politically moderate compared to liberal (OR = 2.671, *p* = 0.048), and being Catholic compared to Protestant (OR = 3.426, *p* = 0.019), and knowledge of SSPs (OR 3.60, *p* = 0.003) were associated with higher odds of naloxone competency.

In regions outside of Omaha and Lincoln (Table 4), knowledge of SSPs (OR 1.813, *p* = 0.017), and higher levels of education compared to no college are associated with higher levels of naloxone familiarity. Knowing someone that experienced an overdose in the past year was associated with lower odds of naloxone familiarity (OR 0.502, *p* = 0.041), as was higher levels of religions attendance (OR 0.911, *p* = 0.041).

However, among people who are familiar with naloxone, those who know someone who experienced an overdose in the past year (OR = 6.06, *p* < 0.001) and having access to illicit opioids or heroin (OR = 3.362, *p* = 0.001), knowledge of an SSP (OR 4.574, *p* < 0.001) was associated with higher odds of naloxone access. Among those familiar with naloxone, being older (OR = 1.021, *p* = 0.037) and having an annual income of more than $100,000 compared to less than $30,000 (OR = 3.39, *p* = 0.005) was associated with higher odds of naloxone access, but income above $100,000 was not positively associated with naloxone familiarity as it was in our statewide model. Knowing someone that experienced an overdose in the past year (OR = 3.56, *p* = 0.014) and having access to illicit opioids or heroin (OR = 2.23, *p* = 0.033) was associated with higher odds of naloxone competency. Perceived community stigma toward people who use opioids was associated with lower odds of naloxone access (OR 0.576, *p* = 0.018) and naloxone competency (OR 0.605, *p* = 0.04).

## Discussion

Our findings corroborate prior studies that reported large gaps in the naloxone treatment cascade between familiarity and possession of naloxone. 74.8% (95% CI [72.0, 77.7]) of respondents knew what naloxone is, but less than a quarter of those familiar knew where to access naloxone or how to use it. Schlosser et al., using Nebraska survey data from 2020, estimated that 68.7% [65.6, 71.7] of respondents were familiar with naloxone, indicating a small but non-trivial increase in population-wide familiarity with naloxone over two years. The estimate of the proportion of respondents aware of where to access naloxone shifts from 15.0% [12.9, 17.5] in 2020 to 18.2 [15.4, 21.0] in 2022. Despite modest growth, naloxone familiarity in Nebraska is nowhere near universal, and rates of naloxone access and competency within the state still have significant room to grow.

Our Nebraska-wide finding of no significant relationship between lifetime illicit opioid use, being close to a past-year overdose, or being close to a person who uses illicit opioids and likelihood of naloxone knowledge is worrying. People in these groups are more likely to be in situations where naloxone use is necessary, but according to our results are not more likely to have naloxone familiarity, access, or competency. In predominantly rural areas, having been close to an overdose in the past year and having access to opioids is associated with higher likelihood of naloxone access and competency, but the lack of association between naloxone knowledge and lifetime opioid use or being close to opioid use remains troubling.

Our study also finds that those who are familiar with SSPs are 129.2% more likely to be familiar with naloxone, 153.3% more likely to know where to access it, and 332.5% more likely to know how to use it. This result corresponds with evidence that using SSPs is correlated with increased naloxone possession [9, 13, 19]. Our result is notable because SSPs are banned in Nebraska, and we are unaware of any unsanctioned distribution efforts [15]. Any benefit emerging from familiarity with SSPs is not due to syringe services provided within Nebraska. Legalizing SSPs, which almost always facilitate naloxone distribution programs [11], in Nebraska may further increase the positive association between familiarity with SSPs and likelihood of naloxone familiarity, access, or competency.

Higher perceived community stigma toward people who use opioids is significantly associated with a decrease in odds of naloxone access and competency. This result aligns with a prior survey of people who inject drugs: Their stigmatization discouraged them from seeking syringes and naloxone, especially in pharmacies [18]. We found that the effect of stigma may extend to people who do not use drugs as well. Community stigma toward people who use heroin, methamphetamines, and cocaine has the opposite effect: Higher perception of stigma is associated with a higher likelihood of naloxone access. To our knowledge, this effect has not been observed in the literature before and is especially notable given its contrast with its association with higher community opioid stigma.

Our results are likely partially capturing differences in stigma toward people by substance type. Heroin, methamphetamines, and cocaine are explicitly illegal, while referring to the category of ‘opioids’ more generally includes legally obtainable prescription opioids. These drug types also contain drastically different cultural connotations: Prescription opioids are pharmaceutical in nature and are linked via media coverage to primarily White suburban and rural communities, while heroin, methamphetamines, and cocaine are linked to racial minorities in urban spaces [16]. Given the stark differences between the way these drug types are perceived, it is likely that perceived stigma toward people who use these drug types would have varied impacts on drug-related knowledge. This may be particularly true in a state like Nebraska which has roughly 45% of the population in two cities and the rest of the state in smaller towns or largely rural areas.

We notably do not find any significant effects linked to gender or race, in contrast with previous studies. Being female compared to being male was positively associated with naloxone familiarity [20] and being White compared to being Black or Hispanic was positively associated with the likelihood of having naloxone training [10].

### Limitations

Our findings have several limitations. First, our measurements of naloxone knowledge are self-reported in a yes/no format rather than asking skill questions (e.g., identifying correct ways to administer naloxone or a location they could obtain naloxone). If respondents exaggerated their knowledge of naloxone, our estimates of overall naloxone knowledge in Nebraska would be biased upward compared to a skill-focused measure of knowledge used in prior studies [8]. Using binary variables to measure naloxone knowledge does not capture nuance such as how many sources of naloxone a respondent could identify or how quickly they could administer naloxone in an emergency. Future research in this area will be needed to build on the present findings.

The Nebraska Annual Social Indicators Survey relies solely on address-based sampling, which has a relatively high response rate and extensive coverage [12]. Despite these benefits, address-based sampling does not reach the unhoused population and can undercount certain demographics including rural areas [5, 17]. To ensure higher levels of participation in rural areas of the state, a stratified sample design was used to include more participants from outside urban areas. The Nebraska age of majority prevents those under 19 years old from completing the survey, excluding 18-year-olds from the sample. This age restriction is a unique feature of Nebraska-specific research.

Another limitation is the inability to examine differences in the naloxone cascade between and within groups of people who are non-White. Less than 10% of sample indicated that they were non-White and no single non-White category had enough participants to compare directly. We therefore compare those who are White and non-Hispanic to everyone else. Although this blurs distinctions among non-White participants, it highlights what has often been an important signifier of how drug policy and prohibition is applied in the USA, if you are White, or not White.

A final limitation is that there remains an unknown potential for non-response bias. With an 18.2% response rate, 81.8% of the sample declined to participate in the survey. Non-response can bias results when non-response is associated with the measures being tested [6]. In this case, if people who know what naloxone is are more or less likely to participate in the survey that could result in our estimates being biased, or if non-response was associated with another outcome. Although potential participants may be reluctant to answer a drug-related study [14], our questions were embedded in an omnibus survey which contained a range of topical questions; although this may have resulted in participants choosing not to answer specific questions, it protects the study from subject-specific refusal to participate at all. Unfortunately, additional tests of non-response bias would require administrative data on the people who did not participate, which is not available in this case.

These restrictions should remain in mind when interpreting our findings.

## Conclusion

Our address-based sample of Nebraska residents shows significant gaps in the naloxone treatment cascade between naloxone familiarity and both access and competency, suggesting the need for increased efforts to increase these factors. There is an alarming lack of association between naloxone knowledge and several features that indicate the respondent is more likely to need to perform a naloxone rescue. While Nebraska currently has naloxone distribution programs in place, further effort is needed to ensure that it is reaching those who need naloxone most. We found that familiarity with syringe service programs was positively associated with naloxone knowledge, and the Nebraska legislature should consider legalizing syringe service programs to further increase this association. Finally, future work should examine the varied impact of perceived substance-specific stigma on naloxone knowledge. Understanding nuances in stigma toward people who use drugs could lead to more efficient and inclusive methods of naloxone distribution in the future.

## Data Availability

The data analyzed during this study—the 2022 Nebraska Annual Social Indicators Survey—will be publicly available upon request to the Bureau of Sociological Research of the University of Nebraska-Lincoln after December 1, 2023.

## Abbreviations

SSPs: Syringe service programs
PWUD: People who use drugs
